# The Paralogous Genes *PDR18* and *SNQ2*, Encoding Multidrug Resistance ABC Transporters, Derive From a Recent Duplication Event, *PDR18* Being Specific to the *Saccharomyces* Genus

**DOI:** 10.3389/fgene.2018.00476

**Published:** 2018-10-15

**Authors:** Cláudia P. Godinho, Paulo J. Dias, Elise Ponçot, Isabel Sá-Correia

**Affiliations:** ^1^iBB-Institute for Bioengineering and Biosciences, Department of Bioengineering, Instituto Superior Técnico, Universidade de Lisboa, Lisbon, Portugal

**Keywords:** ABC transporters, *PDR18* and *SNQ2*, phylogenetic and genomic neighborhood analyzes, comparative genomics and evolution, multidrug resistance

## Abstract

Pleiotropic drug resistance (PDR) family of ATP-binding cassette (ABC) transporters play a key role in the simultaneous acquisition of resistance to a wide range of structurally and functionally unrelated cytotoxic compounds in yeasts. *Saccharomyces cerevisiae* Pdr18 was proposed to transport ergosterol at the plasma membrane, contributing to the maintenance of adequate ergosterol content and decreased levels of stress-induced membrane disorganization and permeabilization under multistress challenge leading to resistance to ethanol, acetic acid and the herbicide 2,4-D, among other compounds. *PDR18* is a paralog of *SNQ2*, first described as a determinant of resistance to the chemical mutagen 4-NQO. The phylogenetic and neighborhood analysis performed in this work to reconstruct the evolutionary history of *ScPDR18* gene in Saccharomycetaceae yeasts was focused on the 214 Pdr18/Snq2 homologs from the genomes of 117 strains belonging to 29 yeast species across that family. Results support the idea that a single duplication event occurring in the common ancestor of the *Saccharomyces* genus yeasts was at the origin of *PDR18* and *SNQ2*, and that by chromosome translocation *PDR18* gained a subtelomeric region location in chromosome XIV. The multidrug/multixenobiotic phenotypic profiles of *S. cerevisiae pdr18*Δ and *snq2*Δ deletion mutants were compared, as well as the susceptibility profile for *Candida glabrata snq2*Δ deletion mutant, given that this yeast species has diverged previously to the duplication event on the origin of *PDR18* and *SNQ2* genes and encode only one Pdr18/Snq2 homolog. Results show a significant overlap between ScSnq2 and CgSnq2 roles in multidrug/multixenobiotic resistance (MDR/MXR) as well as some overlap in azole resistance between ScPdr18 and CgSnq2. The fact that ScSnq2 and ScPdr18 confer resistance to different sets of chemical compounds with little overlapping is consistent with the subfunctionalization and neofunctionalization of these gene copies. The elucidation of the real biological role of *ScSNQ2* will enlighten this issue. Remarkably, *PDR18 i*s only found in *Saccharomyces* genus genomes and is present in almost all the recently available 1,000 deep coverage genomes of natural *S. cerevisiae* isolates, consistent with the relevant encoded physiological function.

## Introduction

Several ATP-binding cassette (ABC) transporters that catalyze the ATP-dependent active solute transport across cell membranes in yeasts are associated with multidrug/multixenobiotic resistance (MDR/MXR) (Jungwirth and Kuchler, 2006; Monk and Goffeau, 2008; Piecuch and Obłak, 2014). Although these transporters are usually considered drug/xenobiotic pumps, evidence is arising supporting the idea that their involvement in MDR/MXR may result from their specific and, in general, not yet determined biological role in the active transport of physiological substrates (Prasad and Panwar, 2004; Cabrito et al., 2011; Prasad et al., 2016; Godinho et al., 2018). Moreover, the presence of a large number of ABC transporters involved in MDR/MXR in the genomes of yeasts and other organisms, from bacteria to man, also strongly suggests that these transporters may play important physiological roles even in the absence of the cytotoxic compounds to which they confer resistance. For example, the yeast ABC-MDR/MXR family of transporters that includes the Pleiotropic Drug Resistance (PDR) transporters, perform endogenous activities beyond their proposed role as drug exporters, in particular as lipid transporters (Balzi and Goffeau, 1995; Borst et al., 2000; Pomorski et al., 2004; Panwar et al., 2008; Van Meer et al., 2008; Prasad et al., 2016). *Saccharomyces cerevisiae* genome encodes 10 PDR proteins: Adp1, Aus1, Pdr5, Pdr10, Pdr11, Pdr12, Pdr15, Pdr18, Snq2, and YOL075c (Decottignies and Goffeau, 1997; Paulsen et al., 1998; Paumi et al., 2009). A combined phylogeny and neighborhood analysis of the evolution of these ABC transporters in nine yeast species belonging to the subphylum Saccharomycotina has shown that Pdr18 is a paralog of Snq2 and that *SNQ2* and *PDR18* genes reside in unshared chromosomal environments (Seret et al., 2009). However, the small number of yeast species genomes available when this study was performed did not allow a firm conclusion concerning the hypothesized gene duplication event at the origin of these two PDR gene sub-lineages. In fact, it was doubtful whether the duplication event remounted to the whole genome duplication (WGD) event or if it was an independent event that occurred post-WGD (Seret et al., 2009).

The *S. cerevisiae* plasma membrane transporter Pdr18 was described as a MDR/MXR determinant required for ergosterol transport at the plasma membrane level (Cabrito et al., 2011; Teixeira et al., 2012; Godinho et al., 2018). Pdr18 expression was found to lead to increased yeast tolerance to the herbicides 2,4-dichlorophenoxyacetic acid (2,4-D), 2-methyl-4-chlorophenoxyacetic acid (MCPA), and barban, the agricultural fungicide mancozeb, the metal cations Zn^2+^, Mn^2+^, Cu^2+^, and Cd^2+^ (Cabrito et al., 2011) and to ethanol (Teixeira et al., 2012) and acetic acid (Godinho et al., 2018). The involvement of Pdr18 in the maintenance of yeast plasma membrane ergosterol content under 2,4-D or acetic acid stresses was related with its role as a determinant of resistance to multiple stresses in yeast (Cabrito et al., 2011; Teixeira et al., 2012; Godinho et al., 2018). A coordinated response involving the transcriptional activation of *PDR18* and several ergosterol biosynthetic genes was found to occur in response to acetic acid stress, strongly suggesting the involvement of Pdr18 in ergosterol homeostasis in stressed yeast cells (Godinho et al., 2018). Moreover, the proposed role for Pdr18 in ergosterol homeostasis was demonstrated to be important to counteract acetic acid-induced decrease of plasma membrane lipid order, increase of plasma membrane non-specific permeability and decrease of transmembrane electrochemical potential (Godinho et al., 2018).

The *PDR18* paralog gene *SNQ2* was first described based on its involvement in yeast resistance to the chemical mutagens 4-nitroquinoline 1-oxide (4-NQO) and triaziquone (Servos et al., 1993). Later, several other publications extended the range of compounds to which *SNQ2* expression confers increased tolerance in yeast (Servos et al., 1993; Hirata et al., 1994; Mahé et al., 1996a,b; Miyahara et al., 1996; Kolaczkowski et al., 1998; Ververidis et al., 2001; Piper et al., 2003; Wehrschütz-Sigl et al., 2004; van Leeuwen et al., 2012; Ling et al., 2013; Nishida et al., 2013; Snider et al., 2013; Tsujimoto et al., 2015). Although no role in lipid homeostasis was demonstrated for Snq2 transporter, it was shown that Snq2 is involved in the alleviation of estradiol toxicity in *S. cerevisiae* (Mahé et al., 1996a). For this reason, it was hypothesized that Snq2 could also have affinity for lipid transport, especially for the estradiol structurally related molecule ergosterol (Mahé et al., 1996a; Kuchler et al., 1997).

Gene duplication is considered to be one of the most important forces driving the evolution of genetic functional innovation and genes encoding membrane transporters are one of the functional gene categories that exhibit high number of duplication events (Ohno, 1970; Zhang, 2003; Taylor and Raes, 2004). In yeast, for example, this is the case of genes encoding proteins of the Major Facilitator Superfamily (MFS) of transporters (Dias et al., 2010; Dias and Sá-Correia, 2013, 2014). Also, transporters from the PDR family were involved in multiple gene duplications and gene losses occurring during their evolutionary history (Seret et al., 2009; Kovalchuk and Driessen, 2010). After gene duplication, it is possible the occurrence of the inactivation of one of the copies (pseudogenization), the maintenance of the two copies (dosage effect), the adoption of part of the function or of the expression pattern of their parental gene (subfunctionalization), or the acquisition of a related or new function (neofunctionalization) (Zhang, 2003; Conant and Wolfe, 2008). The duplicate genes are maintained in the genome depending upon their function, mode of duplication, expression rate and the organism taxonomic lineage (Taylor and Raes, 2004).

To better understand the duplication event that gave rise to the *PDR18* and *SNQ2* paralog genes, the evolutionary history of *PDR18* was reconstructed in this work by combining phylogenetic tree building methodology with gene neighborhood analysis in 117 strains genomes belonging to 29 species across the Saccharomycetaceae family. A systematic multidrug/multixenobiotic phenotypic profiling of *S. cerevisiae* deletion mutants for *PDR18* or *SNQ2* genes was also performed. Given that the genomes of the post-WGD *Candida glabrata* species encode only one Pdr18/Snq2 homolog (*CgSNQ2*) the susceptibility profiling for the *Cgsnq2*Δ deletion mutant was also examined to get additional insights into the functional divergence of *S. cerevisiae* Pdr18 and Snq2 and the common ancestral gene on the origin of the post-WGD single duplication event.

## Materials and Methods

### Identification of the Homologs of *S. cerevisiae* Pdr18 and Snq2 Proteins in Hemiascomycete Yeast Genomes

A total of 1,110,525 Open Reading Frames (ORFs) encoded in the genomes of 171 strains belonging to 68 different yeast species of the subphylum Saccharomycotina were retrieved and compiled in a local Genome DB (Dias and Sá-Correia, 2013, 2014). A second in-house database built in this work, BLASTp DB, comprises the output values of the blastp algorithm (Altschul et al., 1990) for each pairwise entry of all possible combinations between the translated ORFs compiled in the Genome DB, including length of the alignment, e-value, percentage of identity and similarity, and alignment score. The blastp algorithm used a gapped alignment with the following parameters: open gap (-1), extend gap (-1), threshold for extending hits (11), and word size (3). This approach generated a total of 328 million pairwise alignments. The ORFs encoding PDR proteins were identified through the adoption of a network traversal strategy considering the whole set of blastp pairwise relationships as a network that was subsequently traversed at a range of different *e*-value thresholds (Dias and Sá-Correia, 2013, 2014; Palma et al., 2017). The *S. cerevisiae* Pdr18 was selected to represent the PDR *sensu stricto* (Seret et al., 2009) transporters and was used as a starting node for network traversal. The *S. cerevisiae* Adp1 and Yol075c were selected to represent the PDR *sensu lato* transporters (Seret et al., 2009) and also used as starting nodes in independent network traversals. The disjoint sets of translated ORFs obtained from these three traversals were merged. The amino acid sequences of these ORFs were analyzed for potential false positive members of the PDR protein family, protein fragments and/or frameshifts. The remaining amino acid sequences were aligned using MUSCLE software (Edgar, 2004). Subsequently, the protdist and neighbor algorithms made available by the PHYLIP software suite (Felsenstein, 1989) were used to construct a preliminary phylogenetic tree. Using as reference the cluster of residence of the Snq2 and Pdr18 proteins, the branch comprising the homologs of these two *S. cerevisiae* proteins in this tree was identified. For species abbreviation a four letters code is used, composed by the first two letter of the genus and species. The number displayed after the first four letters is used to abbreviate the strain name when the genome of more than one strain from a given species was examined. To standardize the annotation used, translated ORFs are represented by small letters.

### Phylogenetic Analysis and Tree Construction

The MUSCLE software suite (Edgar, 2004) was used to build a multiple alignment of the amino acid sequences of the Snq2 and Pdr18 proteins encoded in Saccharomycetaceae yeasts that was analyzed using the Jalview 2.9 software suite (Clamp et al., 2004; Waterhouse et al., 2009). The processing of these sequences did not involve any step of masking or trimming. The “read.fasta” and “write.nexus.data” functions made available by the seqinr 3.4-5 (Charif et al., 2007) and by the ape 4.1 R packages (Paradis et al., 2004; Popescu et al., 2012), respectively, were used to convert the multiple alignment in fasta format into a nexus file that was subsequently fed into MrBayes 3.2.6 (Huelsenbeck and Ronquist, 2001; Ronquist and Huelsenbeck, 2003), a Bayesian Markov chain Monte Carlo (MCMC) package for phylogenetic analysis. The Message Passing Interface (MPI) version of MrBayes (Altekar et al., 2004) was used to speed up phylogeny computation using Metropolis coupling of MCMC sampling. The MCMC simulations used 100,000 generations, coupling one “cold” chain together with nine heated chains. Two independent runs of MCMC sampling (each started from two distinct random trees) confirmed parameter convergence of the posterior probability distribution. The option of estimating the fixed-rate amino acid prior model made available by MrBayes was used, allowing the MCMC sampler to explore all of the nine available models by regularly proposing new ones (upon parameter convergence, each model contributes to the results in proportion to its posterior probability) and rate variation over sites was assumed to follow a gamma distribution. The remaining MrBayes MPI parameters were set to default values. The PhyML 3.0 software suite (Guindon et al., 2010) was used to construct a Maximum Likelihood (ML) derived phylogenetic tree to confirm the gathered results. The PhyML parameters and models used as input were the default ones, with exception of the method for searching the optimal phylogenetic tree, where the Subtree Pruning and Regrafting (SPR) were used as algorithm instead of Nearest Neighbor Interchange (NNI). The phylogenetic trees obtained were analyzed using the visualization software Dendroscope 3.5.7 (Huson et al., 2007; Huson and Scornavacca, 2012). The MrBayes clade credibility score and PhyML bootstrapping values calculated for each internal node of the Bayesian and ML trees, respectively, were inspected using either the FigTree 1.4.3 software suite^1^ or PhyloTree 0.1 package^2^ installed in Cytoscape 2.8.3 (Shannon et al., 2003). The bootstrap score measures the confidence level of each clade in the consensus phylogenetic tree by repeated sub-sampling data from the original data set and determining the empirical frequency that each of these clades have in the whole set of phylogenetic trees originated from these sub-samples. The clade credibility score measures the posterior probability of each clade in the consensus phylogenetic tree by determining the frequency that each of these clades has in the whole set of phylogenetic tree sampled using the estimated parameter values.

### Gene Neighborhood Analysis

A chromosome block of 30 neighboring genes, 15 on each side of the pair of homologous genes under analysis, was selected to assess the conservation of the chromosome region where the members of the Snq2/Pdr18 subfamily reside (Seret et al., 2009; Dias et al., 2010). Scripting in the R language was used to retrieve 15 neighbor genes on each side of the query genes as well as the corresponding sequence clustering classification from Genome DB. The classification of each of the 30 genes neighboring each query gene was obtained using a conservative blastp *e*-value of E-50 to limit the number of false positive sequences gathered together with true cluster members. When dubious synteny connections between genes needed corroboration, the amino acid sequence clustering was performed at a less restrictive *e*-value threshold of E-40. The existence of synteny between query genes was verified through the analysis of network topology (number of shared neighbor pairs) and the biological information associated with the corresponding edges. Three sources of biological information were used as independent evidence confirming the strength of the synteny between members of the Snq2 and Pdr18 protein subfamily (Dias et al., 2010): (1) distance of the neighbors in relation to the query genes, (2) similarity of the amino acid sequences of the shared neighbors, and (3) total number of members comprised in the cluster of amino acid sequence to which the homologous neighbors belong to; sequence clusters comprising a small number of members are more reliable as synteny evidence since the probability that two homologous neighbors being in the vicinity of two query genes by chance is small.

### Susceptibility Phenotypes of *S. cerevisiae pdr18*Δ and *snq2*Δ and *C. glabrata snq2*Δ Deletion Mutants

#### Strains, Media, and Growth Conditions

*S. cerevisiae* BY4741 (*MATa*, *his3*Δ*1*, *leu2*Δ*0*, *met15*Δ*0*, *ura3*Δ*0*) and the derived deletion mutants *pdr18*Δ and *snq2*Δ were obtained from EUROSCARF collection. *C. glabrata* BPY55 (clinical isolate) and the derived deletion mutant *snq2*Δ built using the SAT1 flipper system were kindly provided by Professor Dominique Sanglard, Institut de Microbiologie, Centre Hospitalier Universitaire Vaudois (CHUV), Lausanne, Switzerland.

Cultivation of *S. cerevisiae* strains was performed in MM4 medium, containing 1.7 g/L yeast nitrogen base without amino acids and ammonium sulfate (Difco, Detroit, MI, United States), 20 g/L glucose (Merck, Darmstadt, Germany), 2.65 g/L (NH_4_)_2_SO_4_ (Panreac AppliChem, CT, United States), 20 mg/L L-methionine, 20 mg/L L-histidine (both from Merck, Darmstadt, Germany), 60 mg/L L-leucine and 20 mg/L L-uracil (both from Sigma, St. Louis, MO, United States). *C. glabrata* strains were cultivated in MM medium, with the composition of MM4 medium, without supplementation with amino acids and uracil. YPD medium contained 20 g/L glucose, 20 g/L Bacto^TM^ Peptone and yeast extract (both from BD Biosciences, Franklin Lakes, NJ, United States). Solid media were prepared by the addition of 20 g/L agar (Iberagar, Barreiro, Portugal) to the different liquid media. Media pH were adjusted to 4.5 with HCl. Growth in liquid media was performed at 30°C with orbital agitation (250 rpm).

#### Susceptibility Tests

Growth susceptibility tests of *S. cerevisiae* parental strain BY4741, the corresponding *pdr18*Δ and *snq2*Δ deletion mutants and of *C. glabrata* BPY55 and derived deletion mutant *snq2*Δ to a wide range of growth inhibitory compounds was evaluated by spot assays. Yeast cell suspensions used for the spot assays were prepared from mid-exponential cell cultures grown in liquid media MM4 (*S. cerevisiae*), MM (*C. glabrata*), or YPD (both strains) by harvesting (5,000 rpm, 5 min) and resuspending the cells in sterile ddH_2_O to an OD_600nm_ of 0.25 followed by four serial dilutions of 1:5 each. These cell suspensions were plated as 4 μL spots onto the surface of MM4 (*S. cerevisiae*) or MM (*C. glabrata*) or YPD (both strains) at pH 4.5 solid media, supplemented or not with the toxic compound to be tested. The plates were incubated at 30°C for 72 h and pictures were taken every 24 h. The toxic compounds tested and the selected concentrations for the screening of *S. cerevisiae* and *C. glabrata* strains in MM4 and MM are listed in **Table 1**, and those selected for experiments in YPD are indicated close to the respective pictures. The susceptibility of the deletion mutants was assessed by comparison of their growth performance with the growth performance of the corresponding parental strain in the presence of the different cytotoxic compounds tested. Moderately reduced growth of the deletion mutants compared with the parental strain was classified as minus (-), marked reduced growth as a double minus (--), improved growth as a plus (+) and identical growth as zero (0). Results are representative from three independent experiments.

**Table 1:** Susceptibility of the deletion mutants *pdr18*Δ and *snq2*Δ of *S. cerevisiae* BY4741 and *snq2*Δ of *C. glabrata* BPY55 to a wide range of chemical compounds supplemented in minimal media.

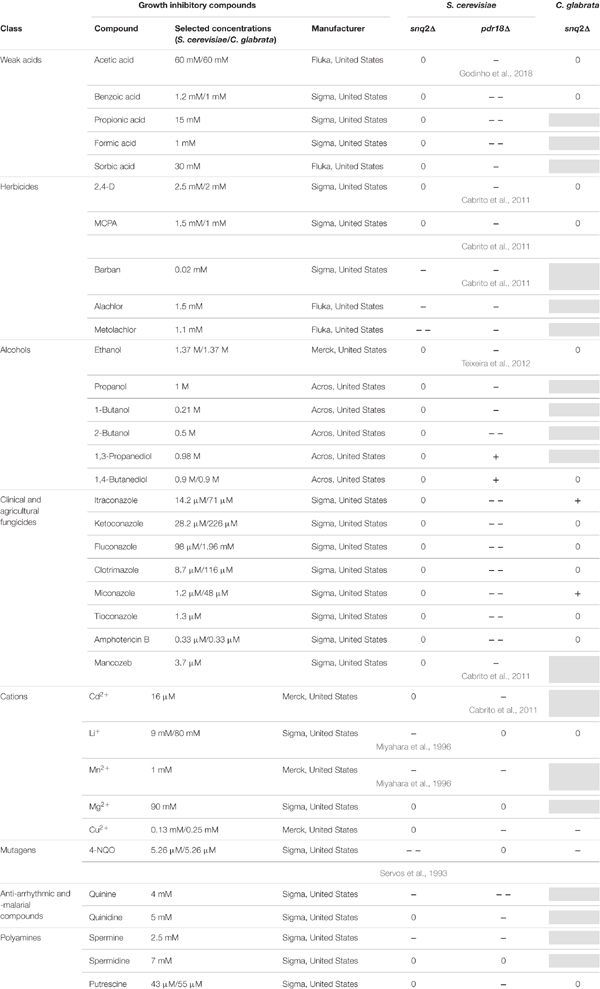

*The phenotypes previously reported and confirmed in our study are identified with the corresponding bibliographic reference. 0: no growth phenotype was identified, - or --: susceptibility phenotype, moderate or marked, respectively, +: resistance phenotype, a gray box indicates that the susceptibility was not tested. Results arise from three independent experiments.*

## Results

### Identification of Snq2 and Pdr18 Proteins Encoded in the Saccharomycetaceae Yeast Genomes

**FIGURE 1:**
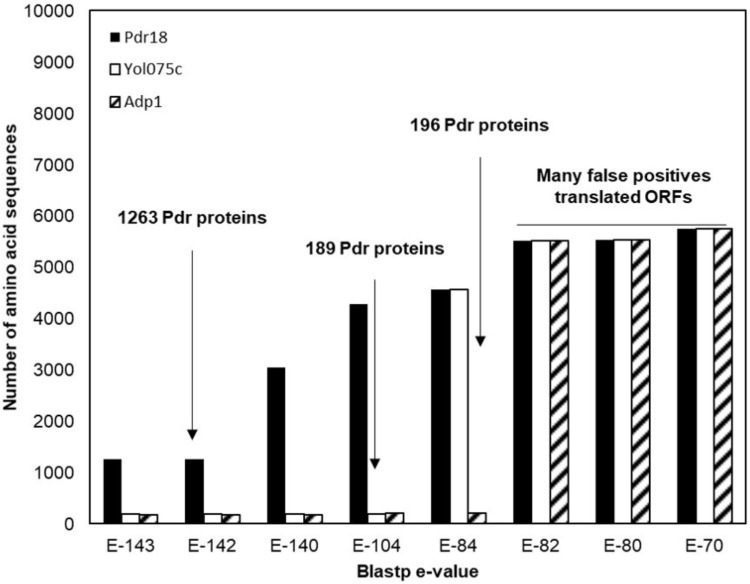
Identification of the PDR proteins encoded in the genomes of 117 strains, belonging to 29 different yeast species belonging to the Saccharomycetaceae family. Plot representing the number of amino acid sequences retrieved after constraining and traversing the pairwise similarity network at different *e*-values taking Pdr18, Yol075c, and Adp1 proteins as starting nodes. The blastp *e*-value used as threshold for network constraining is marked by an arrow.

To identify the PDR proteins encoded in the examined yeast genomes, the network representing the amino acid sequence similarity of the translated ORFs comprised in the Genome DB was traversed at different blastp *e*-value thresholds using as starting nodes *Saccharomyces cerevisiae* proteins Pdr18 [representing the PDR sensu stricto proteins (Seret et al., 2009)] and Yol075c and Adp1 [representing the PDR sensu lato proteins (Seret et al., 2009)]. When Pdr18 was used as starting node the analysis of the protein sets obtained at different blastp e-values indicated that a threshold of E-142 was adequate, allowing the gathering of 1,263 translated ORFs (**Figure 1**). Using a similar approach, a threshold of E-104 and E-84 was found to be adequate for network traversal using Yol075c and Adp1 as starting nodes, allowing the gathering of 189 and 196 translated ORFs, respectively (**Figure 1**). The three disjoint protein sets were merged, giving a total of 1,648 translated ORFs homologous to the yeast PDR proteins in yeast strains belonging to the subphylum Saccharomycotina. This set included members of another family of transporters (hexose transporters) that were manually removed. The corresponding amino acid sequences were aligned using MUSCLE software and the protdist and neighbor algorithms made available by the PHYLIP software suite were used to construct a preliminary phylogenetic tree. Using as reference the cluster of residence of the Snq2 and Pdr18 proteins, the phylogenetic branch comprising the homologs of these two *S. cerevisiae* proteins was identified. The comparison of the PDR protein set gathered in this study with those reported before encoded in the genomes of 10 yeast species (Seret et al., 2009) confirmed the co-clustering of the homologs of *S. cerevisiae* Snq2/Pdr18 proteins in a single branch of the phylogenetic tree. However, since it is well known that translated ORFs residing in long phylogenetic branches may correspond to protein fragments or sequence frameshifts, the blastp algorithm was used to test ORFs with dubious sequence similarity. After determining the homology of each of these proteins selected for blastp testing, a total of 214 translated ORFs, encoded in the Saccharomycetaceae yeast genomes examined, showing strong amino acid sequence similarity to the *S. cerevisiae* Snq2/Pdr18 transporters, were retained for further analysis (**Table 2** and **Supplementary Table S1**).

**Table 2:** Saccharomycetaceae yeast strains examined in this work, and the number of Snq2/Pdr18 homologs, Snq2 orthologs, and Pdr18 orthologs identified.

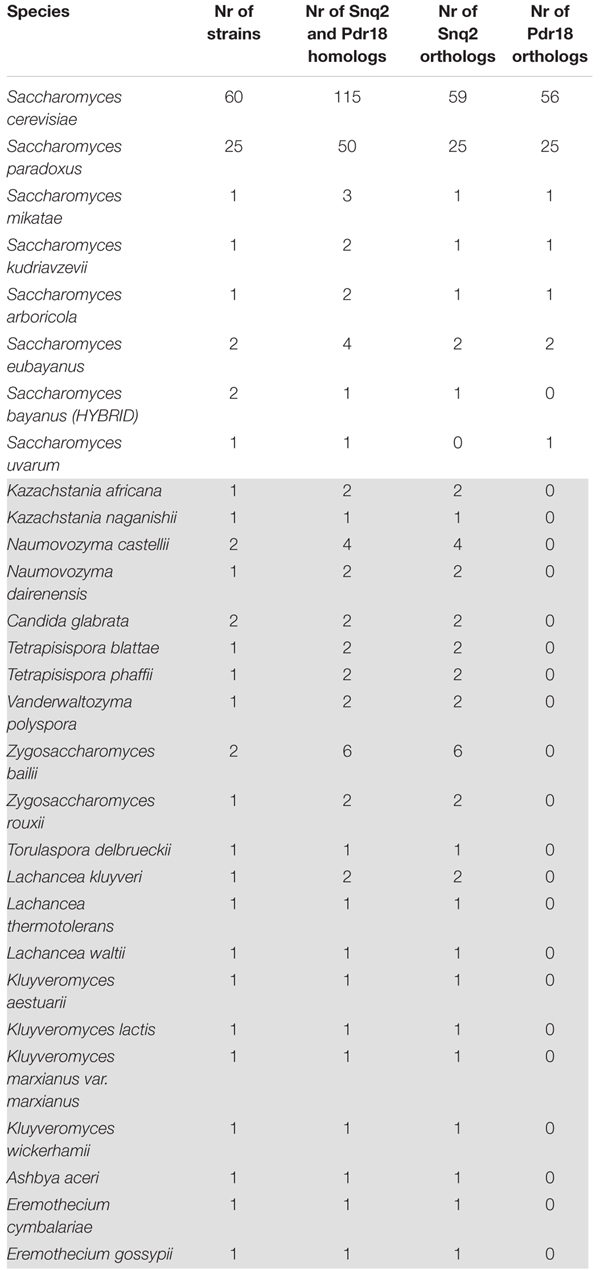

*Pre-whole genome duplication (WGD) species are highlighted in gray. A detailed list of the strains analyzed, number of Pdr18 and Snq2 proteins and sources of genome information and annotation tools is provided as **Supplementary Table S1**.*

### Phylogenetic Analysis of Snq2 and Pdr18 Proteins

**FIGURE 2:**
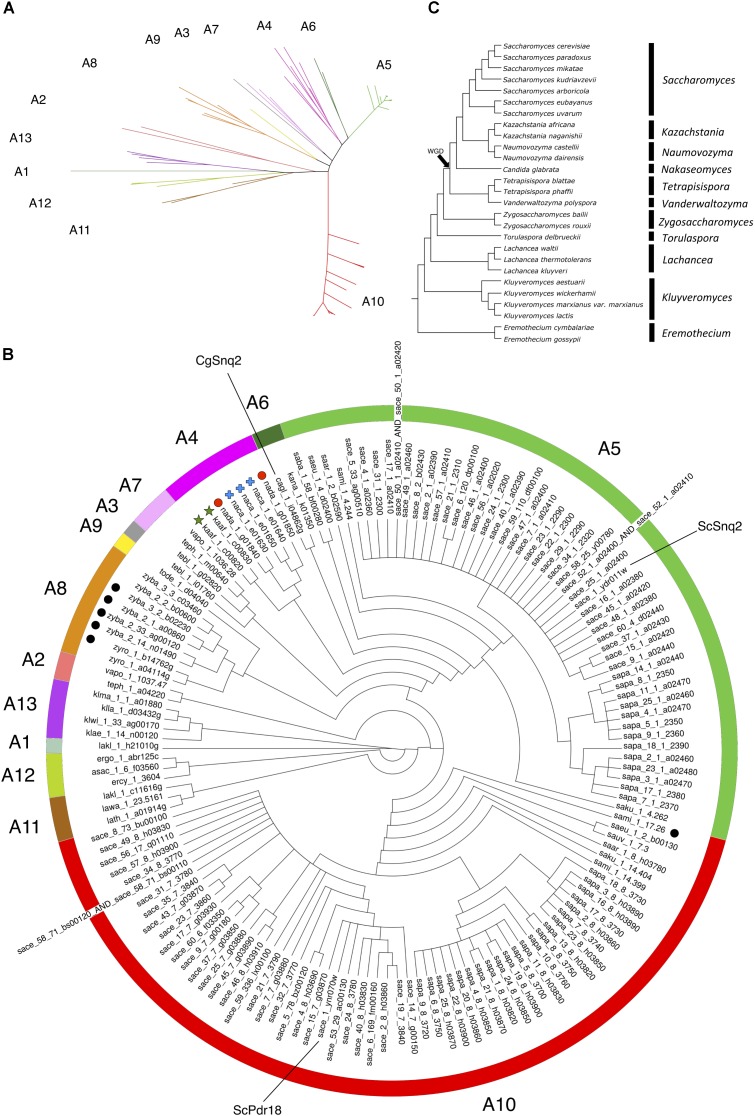
Phylogenetic tree of the Pdr18 and Snq2 homologs encoded in the genome sequences of 117 strains of 29 yeast species belonging to the Saccharomycetaceae family. **(A)** Radial phylogram showing the amino acid sequence similarity distances between the protein homologs of Pdr18 and Snq2. **(B)** Circular cladogram showing the tree topology. Singleton genes were identified with graphical labeling of the translated ORFs using black circles, and tandemly duplicated genes were identified with graphical labeling of the translated ORFs using green stars, red circles and blue crosses. The translated ORF lakl_1_h21010g was chosen as outgroup due to the strong dissimilarity of its amino acid sequence. **(C)** Schematic phylogenetic tree of the species (adapted from Kurtzman and Robnett, 2003).

For the phylogenetic analysis of Snq2 and Pdr18 proteins, more than one strain from a number of yeast species were used (60 strains for *S. cerevisiae*, 25 for *Saccharomyces paradoxus*, 2 for *Saccharomyces eubayanus*, 2 for *Saccharomyces bayanus*, 2 for *Naumovozyma castellii*, 2 for *Candida glabrata*, and 2 for *Zygosaccharomyces bailii*). All the repeated sequences were removed from the protein dataset, leaving just a representative member of each species. This resulted in 146 unique translated ORFs encoded in the Saccharomycetaceae yeast genomes that were used to construct a phylogenetic tree for Pdr18 and Snq2 proteins. For this, the 146 unique translated ORFs were aligned and the corresponding phylogenetic tree constructed using the MrBayes software suite (**Figure 2**). After assuring that all model parameters had converged, the corresponding consensus Bayesian phylogenetic tree was retained for further analysis. Because MrBayes software suite allowed model jumping between nine different fixed-rate amino acid models, after parameter convergence the analysis of results showed that the model Wag (Whelan and Goldman, 2001) has a contribution of 100% to the posterior probability, meaning that this model became dominant over the remaining ones during the MCMC simulation. The translated ORF lakl_1_h21010g was selected as root of the phylogenetic tree because it comprised the most divergent amino acid sequence present in the protein dataset. Complementing the Bayesian approach adopted for the construction of the phylogenetic tree, the PhyML software suite was also used to obtain a ML derived tree (not shown). The analysis of the clade credibility score and bootstrap values obtained for each internal node of the Bayesian and ML trees, respectively, indicates that the two distinct statistical approaches generated similar trees. Due to their similarity, the analysis presented in the manuscript is solely based on the Bayesian tree (**Figure 2**).

The analysis of (i) the tree topology, (ii) the clade credibility score of the branches, and (iii) the phylogenetic distances separating the 146 unique translated ORFs led to the proposal of dividing the tree into thirteen clusters, labeled from A1 to A13 (**Figures 2A,B**). The Snq2 and Pdr18 homologs encoded in *Lachancea*, *Kluyveromyces*, and *Eremothecium* species occupy a basal position in this phylogenetic tree (**Figure 2B** – clusters A1, A11, A12, and A13). On the other hand, the early divergence of the Snq2 and Pdr18 homologs encoded in the genomes of the pre-WGD *Zygosaccharomyces* and *Torulaspora* species is not observed (**Figure 2B** – clusters A8 and A9). All translated ORFs showing strong amino acid sequence similarity to the *S. cerevisiae* Snq2 or Pdr18 proteins are only encoded in the genomes of yeast species classified in the *Saccharomyces* genus. The translated ORFs showing strong amino acid sequence similarity to either *S. cerevisiae* Snq2 or Pdr18 proteins are divided in two distinct branches of this phylogenetic tree, residing in the clusters A5 and A10, respectively (**Figure 2B**). The analysis of the phylogenetic tree also indicates that the Snq2 homologs cagl_1_i04862g and kana_1_k01350 also share strong sequence similarity (cluster 6, 74.5% and 85.9% of identity and similarity, respectively). The percentage of identity and similarity shown between these Snq2/Pdr18 homologs is unexpectedly high suggesting that a lateral gene transfer event to an ancestral strain of *K. naganishii* species might have mediated the acquisition of the Snq2/Pdr18 homolog encoded in this species. The homologs of the Snq2 and Pdr18 proteins encoded in yeast species of the remaining post-WGD taxonomic genera (*Kazachstania*, *Naumovozyma*, *Tetrapisispora*, and *Vanderwaltozyma*) reside in the phylogenetic clusters A2, A3, A4, and A7 (**Figure 2B**).

### Gene Neighborhood Analysis of Pdr18 and Snq2 Orthologs in Saccharomycetaceae Yeasts

Gene neighborhood analysis of *S. cerevisiae SNQ2* and *PDR18* homolog genes encoded in the examined Saccharomycetaceae yeast strains genomes was performed. This study, together with the phylogenetic analysis, is useful to contribute to the elucidation of their ortholog/paralog status.

**FIGURE 3:**
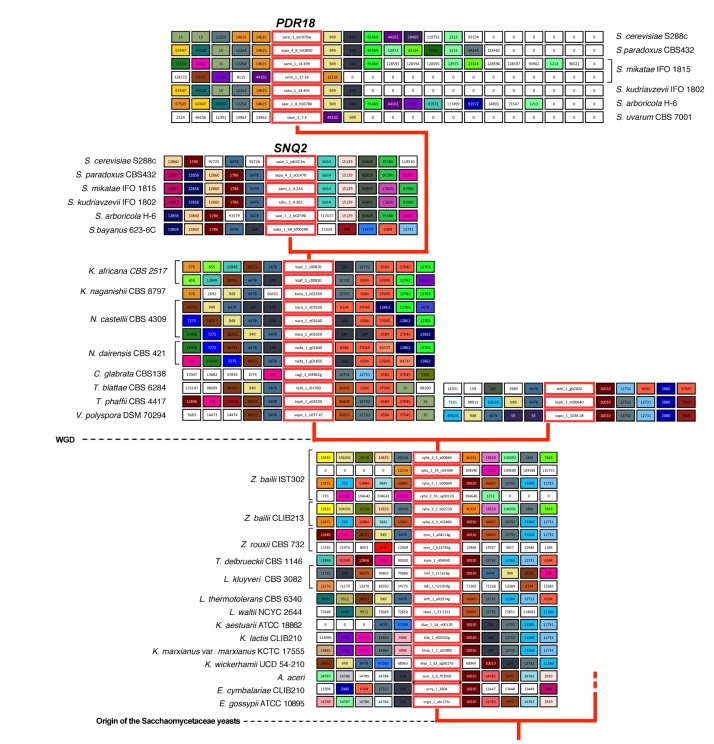
Gene neighborhood of *SNQ2* and *PDR18* genes and corresponding homologs encoded in the genomes examined. Genes in the central boxes represent *SNQ2*, *PDR18* and their orthologs. Adjacent boxes represent gene neighbors and homologous neighbors are highlighted in the same color. A white box represents genes with no homologous neighbors in the represented chromosome region and white boxes with a zero represent the end of the contig/chromosome. The synteny was assessed with 15 neighbors on each side but this representation was truncated to five neighbors. In the case of *PDR18* orthologs, the gene neighborhood data was not truncated in one of the sides to highlight the sub-telomeric position occupied by these genes.

**FIGURE 4:**
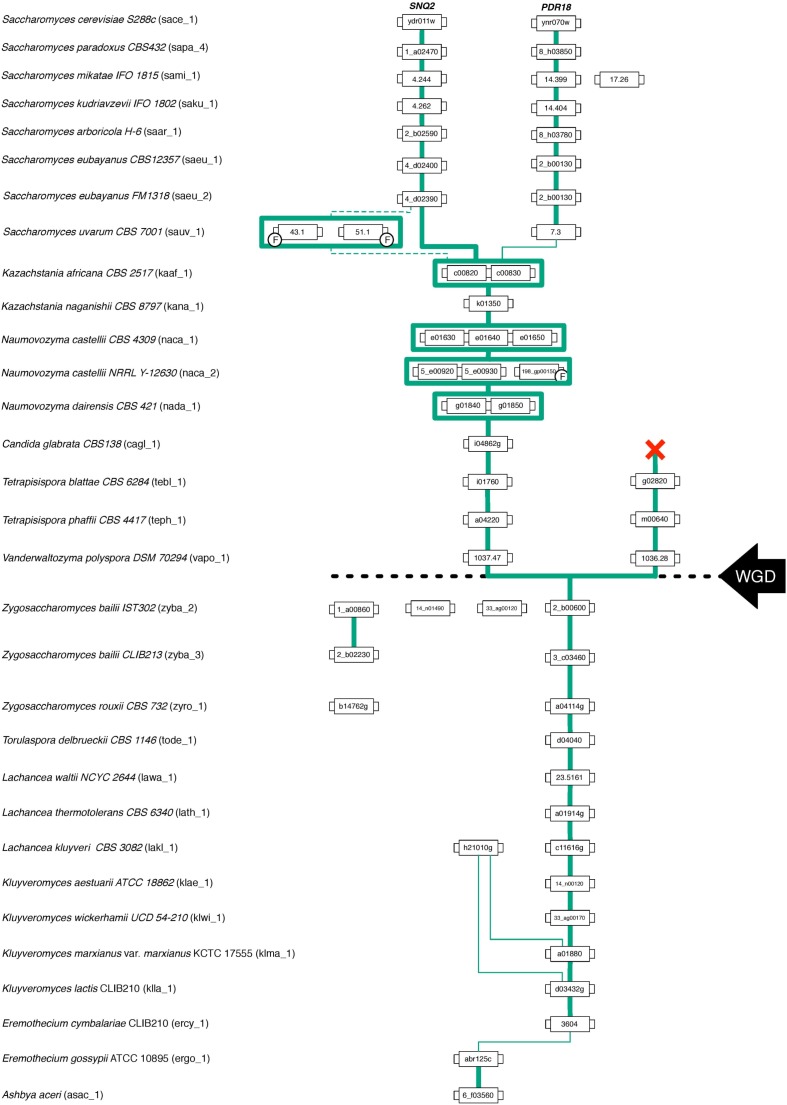
Gene lineage comprising the homologs of *S. cerevisiae PDR18* and *SNQ2* genes encoded in the Saccharomycetaceae species examined. Each box represents a gene and the lines connect genes sharing common neighbors. F indicates that the corresponding gene was classified as a fragment. Line thickness represents the strength of synteny between genes. The black dashed line marks the point in time where the Whole Genome Duplication (WGD) event occurred.

Results show that for the pre-WGD yeasts species of *Torulaspora*, *Lachancea*, *Kluyveromyces*, and *Eremothecium* genera there is a single lineage, with genes sharing strong synteny (**Figures 3**, **4**). The main exception to this rule is the *Lachancea kluyveri* CBS 3082 strain because, in addition to the gene lakl_1_c11616g residing in the above mentioned conserved chromosome environment, this yeast strain also encodes one singleton gene, lakl_1_h21010g, sharing very weak synteny with *Kluyveromyces lactis* and *Kluyveromyces marxianus* var. marxianus genes (**Figure 3**). The pre-WGD yeast strains of the *Zygosaccharomyces* genus, *Z. rouxii* CBS 732 and *Z. bailii* CLIB213, both encode two *SNQ2*/*PDR18* homolog genes (**Figure 3**). However, *Z. bailii* IST302 encodes two additional *SNQ2*/*PDR18* homolog genes, zyba_2_14_n01490 and zyba_2_33_ag00120, lacking common neighbors with the remaining *SNQ2*/*PDR18* homolog genes in the Saccharomycetaceae strains genomes examined (**Figure 3** and **Supplementary Figure S1**). For this reason, these two genes were considered singletons. The high amino acid sequence identity shared by these two genes suggests that they are a paralog pair originated in a duplication event that occurred recently in the evolution of *Zygosaccharomyces* species.

After the WGD event, the above described single gene lineage gave rise to two gene sub-lineages and the chromosome regions where these genes reside in pre- and early-divergent post-WGD yeast species are conserved (**Figure 3**). For instance, the analysis of the neighborhood of the pre-WGD *Zygosaccharomyces rouxii* gene zyro_1_a04114g and of the post-WGD species *Vanderwaltozyma polyspora* gene vapo_1_1037.47 shows the existence of 9 common neighboring genes, some of them absent from **Figure 3** but that can be seen in **Supplementary Figure S1**. *Z. rouxii* gene zyro_1_a04114g also shares 9 common neighboring genes with the second gene encoded in *V. polyspora* (vapo_1_1036.28), belonging to the second sub-lineage of ohnolog genes originating from WGD (**Figure 3** and **Supplementary Figure S1**).

The *Candida glabrata* strains’ genomes analyzed only encode one Pdr18/Snq2 homolog gene sharing strong synteny with all the homologs encoded in post-WGD species, from *Naumovozyma dairenensis* to *Kazachstania africana* (**Figure 3** and **Supplementary Figure S1**). This sub-lineage also shows strong synteny with one of the gene sub-lineages encoded in *Saccharomyces* genus species, corresponding to the Sc*SNQ2* orthologs in *Saccharomyces* yeasts, supporting the Sc*SNQ2*-ortholog status for the *SNQ2*/*PDR18* homologs encoded in the post-WGD species, from *C. glabrata* to *S. cerevisiae* (**Figures 3**, **4**). We also hypothesize the loss of the ohnolog gene from the second sub-lineage in the common ancestor of *Saccharomyces*, *Nakaseomyces*, *Kazachstania*, and *Naumovozyma* genera, given that all the encoded *PDR18*/*SNQ2* homologs from *C. glabrata* to *K. africana* appear to be Sc*SNQ2* orthologs.

The gene neighborhood analysis of the *S. cerevisiae SNQ2*/*PDR18* homologs encoded in the genomes of the *Saccharomyce*s genus yeast species *S. paradoxus*, *S. mikatae*, *S. kudriavzevii*, and *S. arboricola*, shows the existence of two gene sub-lineages, one comprising the above described *SNQ2* orthologs and the other comprising *PDR18* orthologs. *PDR18* orthologs occur exclusively in the *Saccharomyces* genus yeast species and the sub-lineage constituted by these orthologs shows a very strong synteny (**Figure 3**). However, the analysis of the gene neighborhood of the *SNQ2* and *PDR18* orthologs in *Saccharomyces* species residing in a basal phylogenetic position could not be performed due to lack of data: the translated ORF encoding the *S. uvarum SNQ2* ortholog, and the *PDR18* ortholog encoded in the genome of *S. bayanus* 623-6C were fragmented into two different contigs of small dimension. Therefore the closely related species *S. eubayanus* (Baker et al., 2015), whose genome had not been at first included in Genome DB, was used instead. The chromosome environment where the *SNQ2* and *PDR18* orthologs reside in the *S. eubayanus* genome was inspected by performing manual blastp pairwise comparisons of the amino acid sequence of the neighboring genes against the full protein set of *S. cerevisiae*. This analysis also showed that *S. eubayanus* CBS12357 and FM1318 strains encode a single *SNQ2* ortholog and a single *PDR18* ortholog sharing strong synteny with the remaining genes within each sub-lineage. These results support the notion that the chromosome environment where *SNQ2* and *PDR18* paralogs reside in the genome of *Saccharomyces* species has been conserved since their appearance in the last common ancestor of the species comprised in this taxonomic genus.

The *S. cerevisiae PDR18* gene resides in the subtelomeric region of chromosome XIV (**Figure 3**), a region poorly conserved throughout Saccharomycetaceae yeasts’ evolution, sharing little synteny with the homologs of the *SNQ2/PDR18* genes encoded in the genomes of yeast species belonging to the other post-WGD taxonomic genera. In fact, the only neighbor in the vicinity of the *PDR18* gene that did not belong to large gene families and is also shared with the *K. naganishii*, *N. castelli* and *N. dairenensis* species is ORF YNR071C, a non-biochemically characterized member of a gene family of aldose 1-epimerases (Li et al., 2013). This ORF and the other *S. cerevisiae* members of this gene family, *GAL10* gene and ORF YHR210C, are comprised in the cluster of amino acid similarity 949 (**Figure 3**). This cluster of amino acid sequence similarity comprises a total of 366 members in the 170 hemiascomycetous strains of the Genome DB (gathered at an *e*-value threshold of E-50). The gene neighborhood analysis showed that the members of this cluster are present in the chromosome environment where Snq2 orthologs from pre- and post-WGD species and Pdr18 orthologs from *Saccharomyces* genus species reside (**Figure 3**).

Although the gene neighborhood results suggest that the gene duplication event giving rise to the *S. cerevisiae SNQ2* and *PDR18* genes occurred in a recent ancestor of the genus *Saccharomyces*, the phylogenetic relationships between the A5 and A10 clades (**Figure 2**) is apparently not consistent with the proposed evolutionary scenario. To clarify this seemingly inconsistency, the amino acid sequences of the orthologs of the *SNQ2*/*PDR18* genes encoded in the post-WGD species (left-sublineage in **Figure 4**) were used to construct a phylogenetic tree using the same initial parameters used in construction of the tree shown in **Figure 2** (**Supplementary Figure S2**). The decision of constructing a new tree was based on the plausible notion that the inclusion of non-orthologous sequences might be introducing “noise” in the multiple alignment of the sequences and, consequently, distorting tree topology and introducing error in the true phylogenetic relationships established between the genes comprised in the WGD left sub-lineage. After parameter convergence, the analysis of this phylogenetic results indicated that the model Jones (Jones et al., 1992) has a contribution of 100% to the posterior probability. In fact, the analysis of this new tree clearly shows that the node joining clades A5 and A10 defines a branch comprising only genes encoded in the *Saccharomyces* species and that the last common ancestor of the homologs of the *SNQ2* and *PDR18* genes coincides with the origin of the *Saccharomyces* genus. The obtained clade credibility score for the orthologs of these two *S. cerevisiae* genes residing together in a single phylogenetic branch of this Bayesian consensus tree was calculated as being 0.6724. These results are consistent with the evolutionary scenario proposed based on the gene neighborhood analysis and confirm the previously proposed existence of a paralogous relationship between the *S. cerevisiae SNQ2* and *PDR18* genes but also establishing that the gene duplication event in its origin occurred more recently than formerly proposed (Seret et al., 2009), presumably coinciding with the first common ancestor of the yeast species comprised in the *Saccharomyces* genus, before the radiation of these species.

### Susceptibility Profiling of *S. cerevisiae snq2*Δ and *pdr18*Δ and *C. glabrata snq2*Δ Deletion Mutants

The post-WGD *C. glabrata* species genome encodes a sole ScSnq2 ortholog, the CgSnq2 (Sanglard et al., 2001). The encoded gene has diverged before the hypothesized duplication event that originated *S cerevisiae PDR18*. For this reason, *C. glabrata* was the selected species to get insights into the functional divergence of the ancestral gene and *S. cerevisiae PDR18* and *SNQ2* genes. Since ScPdr18, ScSnq2, and CgSnq2 were reportedly involved in MDR/MXR, the functional divergence of these proteins was examined by profiling the growth susceptibility of the corresponding deletion mutants against a wide range of cytotoxic compounds, some of them already described as potential substrates for these drug/xenobiotic pumps. The susceptibility of *S. cerevisiae* BY4741-derived *snq2*Δ and *pdr18*Δ deletion mutant strains to a wide range of chemical compounds was screened under identical conditions in minimal medium MM4 (*S. cerevisiae*), ranging from weak acids, alcohols, polyamines, metal cations to herbicides, fungicides and anti-arrhythmic and -malarial compounds (**Table 1**). The deletion of the *SNQ2* gene in *S. cerevisiae* was found to lead to increased susceptibility to 4-NQO, Li^+^ and Mn^2+^, in agreement with previous studies (Servos et al., 1993; Miyahara et al., 1996). Moreover, this systematic screening contributed to extend to the herbicides barban, alachlor, and metolachlor, the anti-malarial anti-arrhythmic quinine and the polyamine spermine the list of toxic compounds to which Snq2 confers protection in *S. cerevisiae* (**Table 1**).

The deletion of the *PDR18* gene was found to render *S. cerevisiae* cells more susceptible toward almost all the compounds tested, contrasting with *SNQ2* whose MDR/MXR spectrum is apparently more limited, the phenotypes only coinciding for 6 of the 35 compounds tested (**Table 1**). In particular, the higher toxic effect of weak acids and of fungicides that target either ergosterol biosynthesis (azoles) or the ergosterol molecule itself (amphotericin B) toward the *pdr18*Δ strain is evident (**Table 1**), consistent with the role described for this ABC transporter in acetic acid resistance and in ergosterol transport at plasma membrane (Cabrito et al., 2011; Teixeira et al., 2012; Godinho et al., 2018).

The expression of *CgSNQ2* gene in *C. glabrata* was found not to confer either protection or increased susceptibility to stress induced by acetic and benzoic acids, the herbicides 2,4-D and MCPA, the alcohols ethanol and 1,4-butanediol, and the polyamine putrescine in minimal medium, similarly to former observations for the expression of *ScSNQ2* in *S. cerevisiae.* This profile is considerably different from that exhibited by *S. cerevisiae* cells devoid of *PDR18*, which were more susceptible to all these compounds, except for 1,4-butanediol, toward which this deletion mutant was more tolerant (**Table 1**). Moreover, susceptibility phenotypes for *Cgsnq2*Δ deletion mutant were detected with toxic concentrations of the mutagen 4-NQO [which is a described putative substrate for *S. cerevisiae* efflux pump Snq2 (Servos et al., 1993)]. However, the susceptibility phenotype for the lithium cation exhibited by *Scsnq2*Δ mutant, was not detected for the *Cgsnq2*Δ mutant (**Table 1**). Moreover, CgSnq2 is apparently a determinant of resistance to Cu^2+^, while no phenotype was found for *Scsnq2*Δ (**Table 1**). The toxic effect of the azole drugs clotrimazole, ketoconazole, and fluconazole, and of amphotericin B was not alleviated by the expression of *CgSNQ2* when tested in minimal medium, consistent with what was observed for *S. cerevisiae ScSNQ*2 expression. Surprisingly, in the presence of itraconazole and miconazole, the deletion of *CgSNQ2* was apparently advantageous (**Table 1**).

**FIGURE 5:**
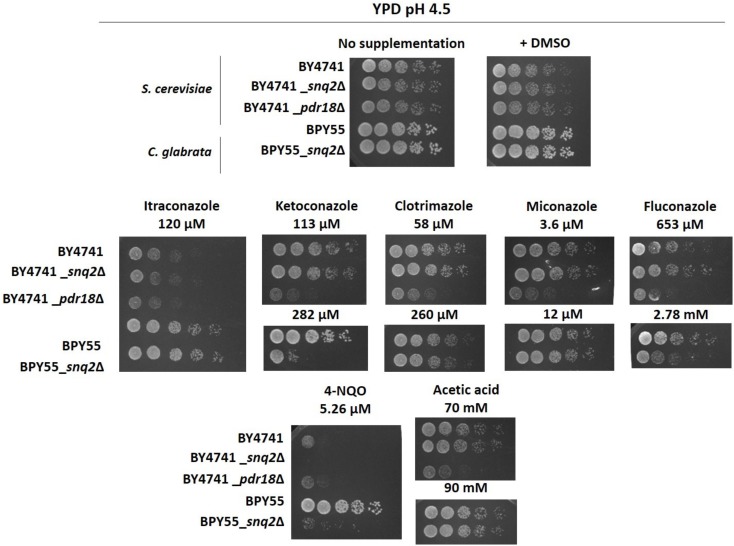
Susceptibility profiling of the *S. cerevisiae pdr18*Δ and *snq2*Δ and of *C. glabrata snq2*Δ deletion mutants to azole drugs, 4-NQO and acetic acid in rich media YPD. Comparison of growth by spot assays of the *S. cerevisiae* parental strain BY4741 and derived *pdr18*Δ and *snq2*Δ, and of *C. glabrata* parental strain BPY55 and derived *snq2*Δ mutant cell suspensions plated in solid YPD media supplemented or not with azole drugs, 4-NQO or acetic acid (at pH 4.5). All pictures were obtained after 24 h of incubation. The results are representative from three independent experiments.

Although the results in **Table 1** suggest that CgSnq2 has no positive effect in *C. glabrata* BPY55 resistance to azoles, a susceptibility phenotype for the BPY55_*snq2*Δ mutant in the presence of the azole drugs fluconazole and ketoconazole was previously reported (Torelli et al., 2008). However, the spot assays performed in the referred study were performed in YPD medium while all the phenotypes described in **Table 1** for *C. glabrata* were performed in minimal medium MM. Therefore, we investigated the susceptibility to azole drugs in rich medium YPD for *C. glabrata* strains, as well as for *S. cerevisiae* strains to confirm results obtained in minimal media MM4 (**Figure 5**). We also included the well-characterized phenotypes for *Scpdr18*Δ and *Scsnq2*Δ in the presence of acetic acid and 4-NQO, respectively, and confirmed that there is no interference of the growth medium used in the phenotypes obtained (**Figure 5**). The higher susceptibility of *Scpdr18*Δ deletion mutant, when compared to the corresponding parental strain, to the azole drugs ketoconazole, clotrimazole, miconazole, fluconazole, and to a lower extent, to itraconazole was confirmed in YPD (**Figure 5**). Also, as found in minimal medium MM4 (**Table 1**), Sc*snq2*Δ shows no susceptibility phenotype in the presence of the azole drugs in YPD (**Figure 5**). In summary, even in YPD media, CgSnq2 is not a determinant of *C. glabrata* tolerance to itraconazole, clotrimazole or miconazole (**Figure 5**), but the phenotypes previously reported for fluconazole and ketoconazole (Torelli et al., 2008) were here confirmed.

## Discussion

The evolutionary history of *Saccharomyces cerevisiae PDR18* gene in Saccharomycetaceae yeasts was reconstructed in this study. Compared with a former analysis based on the genome sequences of only nine yeast species belonging to the *Hemiascomycetes* phylum (Seret et al., 2009), our study took advantage of the increasing number of yeast genomes currently available, and has examined sixteen post-WGD yeast species instead of only two, spanning six different taxonomic genera, instead of only two. This fact allowed the clarification of the evolution of *S. cerevisiae PDR18* and *SNQ2* genes homologs after the WGD event and led us to propose that a single gene loss event has occurred in the last common ancestor of *Nakaseomyces*, *Naumovozyma*, *Kazachstania*, and *Saccharomyces* yeasts. This event explains the “interruption” of one of the post-WGD sub-lineages given that yeast strains of the *Nakaseomyces*, *Naumovozyma*, and *Kazachstania* genera encode only one *PDR18*/*SNQ2* homolog gene with high synteny between them. The probability that the duplication event has occurred during the WGD event is negligible, as it would imply the gene loss in the five yeast species belonging to the *Nakaseomyces*, *Naumovozyma*, and *Kazachstania* genera. Also, the gene neighborhood analysis does not provide support to the second evolutionary scenario, as only one common neighboring gene in these sub-lineages belongs to the similarity cluster 949, which is also present in the chromosome environment of many genes comprised in the other post-WGD sub-lineage proposed to give rise to the duplication event. Altogether, results do not support the ohnolog status for *S. cerevisiae SNQ2* and *PDR18* genes, but instead support the first proposed scenario.

Concerning the point in time where the duplication event originating the *SNQ2* and *PDR18* sub-lineages might have occurred in the evolution of the post-WGD yeast species, it is possible that the *PDR18* gene ancestor was nurtured in one of the tandem repeats encoding *PDR* genes that, with exception of the *K. naganishii* genome, are found in the genomes of all yeast species belonging to the *Kazachstania* and *Naumovozyma* genera. Under this scenario, the amino acid sequence of the ancient gene that gave rise to *PDR18* did not diverge until the last common ancestor in the origin of the *Saccharomyces* genus split apart from the ancestral yeast population giving rise to the *Kazachstania* genus. In the second scenario, the *PDR18* gene ancestral was originated in an independent duplication event not related with the events on the origin of the tandem repeats observed in the genomes of the *Kazachstania* and *Naumovozyma* species. Independently of which of these evolutionary scenarios is true, subsequent genome shuffling and/or other mechanisms of genome evolution should have been responsible for the transposition of the two ancestral genes encoding Pdr18 and the neighboring gene, encoding the aldose 1-epimerase, into a new chromosome environment.

Beside the gene duplication events in the origin of the *SNQ2* and *PDR18* paralog genes, other gene duplication events were identified in the genomes of the protoploid Saccharomycetaceae (pre-WGD). The yeast species belonging the *Zygosaccharomyces* genus and the *Lachancea kluyveri* species encoded more than one Snq2/Pdr18 homolog in their genomes, escaping the typical pattern observed in the majority of the pre-WGD species analyzed in this study, where a sole member of the Snq2/Pdr18 protein subfamily were found encoded in the corresponding genome sequences. Interestingly, the amino acid sequences of the two singletons encoded in the genome of the *Z. bailii* IST302 strain showed the existence of a strong divergence in respect to the amino acid sequences of the Snq2/Pdr18 homologs encoded in the other *Zygosaccharomyces* strains analyzed in this study. This fact and the high amino acid sequence identity shared by these two singletons suggests that these are a paralog pair originated in a duplication event that occurred recently in the evolution of the *Zygosaccharomyces* yeast species. The consultation of the MIPS website comprising the aligning of the presently available genome sequences of yeast strains belonging to the *Zygosaccharomyces* genus showed that these two singleton genes are also absent in the interspecies hybrid Z. bailii ISA1307^3^. This result furthers strengths the hypothesis of a recent origin of these two singletons occurring at the intraspecific level.

Since it was found that *PDR18* is specific for the *Saccharomyces* genus, with very high conservation among the 93 *Saccharomyces* yeast genomes examined, the MDR/MXR profiling of this transporter in *S. cerevisiae* was examined for a wide number of relevant toxic compounds and the possible overlapping of the susceptibility phenotypes exhibited by the *Scpdr18*Δ mutant and the *Scsnq2*Δ mutant with its paralog gene *ScSNQ2* deleted was systematically examined. ScSnq2 was found to confer resistance to a more restricted range of the toxic compounds tested, compared with ScPdr18 with a demonstrated physiological function as plasma membrane transporter in the maintenance of plasma membrane ergosterol content specially under chemical stress. This biological role was related with the decreased levels of stress-induced membrane disorganization and permeabilization and counteracting transmembrane electrochemical potential dissipation (Cabrito et al., 2011; Godinho et al., 2018) and thus with the maintenance under stress of a functional plasma membrane as a selective barrier and a suitable lipid environment for the physiological activity of the embedded proteins (del Castillo Agudo, 1992; Parks and Casey, 1995; Eisenkolb, 2002; Aguilera et al., 2006; Abe and Hiraki, 2009; Caspeta et al., 2014; Kodedová and Sychrová, 2015).

Since the anticipated functional divergence between ScPdr18, ScSnq2 and the ancestral gene on the origin of the duplication event is of relevance to understand the evolutionary process acting on the two duplicate genes, the post-WGD pathogenic species *Candida glabrata* was selected for a systematic analysis of the susceptibility phenotype of the corresponding deletion mutant *Cgsnq2*Δ. Based on the susceptibility assays performed, the sole *SNQ2*/*PDR18* homolog in *C. glabrata* encoding CgSnq2 appeared to be functionally closer to ScSnq2 then to ScPdr18, playing a role in 4-NQO resistance in both species and having no impact in *C. glabrata* tolerance to the weak acids acetic and benzoic, the herbicides 2,4-D and MCPA, the alcohols ethanol and 1,4-butanediol, and the polyamine putrescine, to which *ScPDR18* expression confers tolerance. It is noteworthy that CgSnq2 was previously found to play a role in *C. glabrata* BPY55 tolerance to fluconazole and ketoconazole by susceptibility assays in solid YPD medium (Torelli et al., 2008). This phenotype was confirmed in this study under similar conditions, a fact that could indicate some overlapping of the function associated to CgSnq2 and ScPdr18. However, these phenotypes were not reproduced in minimal media MM, the growth conditions used for the systematic analysis of the phenotypic profiling performed.

The apparent overlapping between ScPdr18 and CgSnq2 role observed in azole resistance in rich media can be related with species-specific adaptation of *C. glabrata* to these fungicides, given that this yeast species is one of the most common in nosocomial fungal infections and that azoles are one of the main families of drugs that are currently being used to treat or prevent fungal infections (Perlroth et al., 2007; Jandric and Schüller, 2011; Roetzer et al., 2011) and that the *C. glabrata* strain used in this work is a highly azole resistant clinical isolate (Torelli et al., 2008). The sensitivity phenotype exhibited by *Cgsnq2*Δ toward Cu^2+^ toxicity might also be related to the fact that BPY55 is a clinical isolate and that adaptation to high Cu^2+^ environmental concentrations is a described determinant for survival of human pathogens (Festa and Thiele, 2012; Samanovic et al., 2012; Chaturvedi and Henderson, 2014; Fu et al., 2014; García-Santamarina and Thiele, 2015).

The fact that *S. cerevisiae* Snq2 and Pdr18 confer resistance to a very different set of chemical compounds with little overlapping, appears to exclude the evolutionary scenario where these highly similar MDR/MXR transporters have been retained in the *S. cerevisiae* genome due to functional redundancy or dosage effect, suggesting as the most consistent scenarios the subfunctionalization and the neofunctionalization of the gene copies. Although subfunctionalization is commonly associated with the mere division of functions of the ancestral protein by the two duplicates, another possible model is that one of the duplicate proteins becomes more efficient at performing one of the original functions of the progenitor gene (Zhang, 2003). Considering the neofunctionalization process, in most of the cases the function adopted by one of the duplicate proteins is a related function rather than an entirely new function (Zhang, 2003; Conant and Wolfe, 2008). Although there are no detailed studies focusing on *SNQ2* expression impact on yeast plasma membrane lipid content and properties, Snq2 was previously found to contribute to alleviate estradiol toxicity in *S. cerevisiae*, a molecule highly similar to ergosterol (Mahé et al., 1996a) and Pdr18 is described to be an ergosterol transporter at the plasma membrane (Godinho et al., 2018). The clarification of whether ScSnq2 and/or CgSnq2 may play some role on lipid homeostasis and of whether ScPdr18 is the result from subfunctionalization by function specialization or whether the biological function acquired by ScPdr18 is totally different from ScSnq2 and CgSnq2, require further work and the elucidation of *SNQ2* biological function.

Pdr18 is encoded in at least 87 out of the 93 genomes from the *Saccharomyces* genus yeasts examined in this study: in 56 out of 60 *S. cerevisiae* genomes, in the 25 *S. paradoxus* genomes, in the genomes of *S. mikatae*, *S. kudriavzevii*, *S. arboricola*, and *S. uvarum*, and in the two *S. eubayanus* genomes. DNA degradation during preparation for genome sequencing is the most plausible explanation for the lack of the PDR18 gene in some of the strains. For example, *PDR18* gene was not found in the genome sequence of *S. cerevisiae* cen.pk113-7d strain since the genome sequencing shows no coverage for the right arm of chromosome XIV, where *PDR18* gene resides (Nijkamp et al., 2012). Moreover, *PDR18* gene was found to be present in the genomes of 964 isolates of more than 1,000 natural *S. cerevisiae* isolates that were a recently examined using deep coverage genome sequencing (Peter et al., 2018). This fact is consistent with the relevant physiological function encoded by *PDR18* in this yeast species (Godinho et al., 2018).

## Data Availability

The dataset generated for this study can be found in TreeBase (http://purl.org/phylo/treebase/phylows/study/TB2:S23282).

## Author Contributions

PD and CG carried out the phylogenetic and gene neighborhood analysis. CG and EP carried out the susceptibility tests. IS-C guided and coordinated this study and together with CG and PD prepared the manuscript. All authors read and approved the final manuscript.

## Conflict of Interest Statement

The authors declare that the research was conducted in the absence of any commercial or financial relationships that could be construed as a potential conflict of interest.

## Footnotes

1 http://tree.bio.ed.ac.uk/software/figtree/

2 http://apps.cytoscape.org/apps/phylotree

3 https://www.helmholtz-muenchen.de/ibis/index.html
