# Supplementary material for: The Paralogous Genes PDR18 and SNQ2, Encoding Multidrug Resistance ABC Transporters, Derive From a Recent Duplication Event, PDR18 Being Specific to the Saccharomyces Genus
Source: Front Genet. 2018 Oct 15;9:476. doi: 10.3389/fgene.2018.00476 (PMC6196229; doi:10.3389/fgene.2018.00476)

Supplementary Material

The paralogous genes *PDR18* and *SNQ2*, encoding multidrug resistance ABC transporters, derive from a recent duplication event, *PDR18* being specific to the *Saccharomyces* genus

Cláudia P. Godinho#, Paulo J. Dias#, Elise Ponçot, Isabel Sá-Correia*

#- These authors have contributed equally to this work

***Correspondence:**
Prof. Dr. Isabel Sá-Correia.
Institute for Bioengineering and Biosciences, Instituto Superior Técnico
Av Rovisco Pais, 1049-001 Lisboa, Portugal
isacorreia@tecnico.ulisboa.pt

Supplementary figure S1. Gene neighborhood of the *ScPDR18* and *ScSNQ2* orthologs encoded in the Saccharomycetaceae species genomes examined. Central boxes represent Sc*PDR18/ScSNQ2* orthologs and the adjacent boxes represent gene neighbours. Homologous neighbours are highlighted in the same colour and identified with the same number. A white box represents genes with no homologous neighbours in the represented chromosome region and white boxes with a zero represent the end of the contig/chromosome.

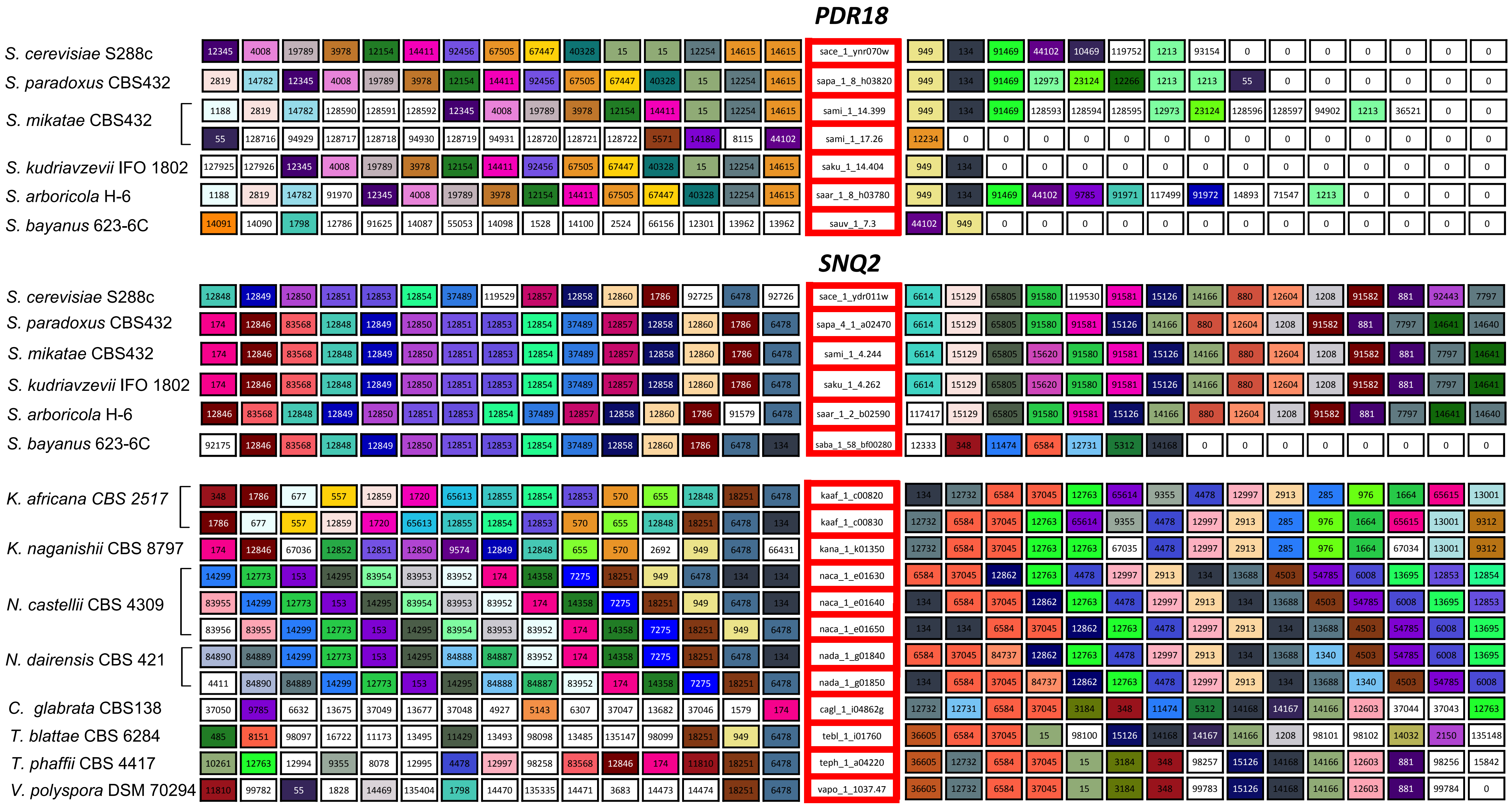

Supplementary figure S1 (continued)

T. blattae CBS 6284	2292	3241	97965	97966	13861	12382	97967	97968	14334	2911	14331	159	265	2989	6478	tebl_1_g02820	10010	12731	6584	2080	37045	7669	3184	12664	6614	348	15129	97969	11474	15620	5312
T. phaffii CBS 4417	12864	135219	12867	12868	1208	963	12871	12872	98916	7161	7161	98915	65620	949	6478	teph_1_m00640	10010	12732	12731	2080	7669	12664	6614	11474	12991	12990	12989	12988	12987	65617	12983
V. polyspora DSM 70294	12867	12868	1208	963	12871	12872	99767	7161	7161	99768	65620	949	6478	55	55	vapo_1_1036.28	10010	12732	12731	2080	7669	9574	12852	12853	4508	1260	99769	12857	12859	12860	557
Z. bailii IST302	13840	20797	135761	635	13838	13837	13836	13835	13834	5643	13833	104391	15510	13829	20246	zyba_2_1_a00860	46332	13828	104392	1464	7843	13826	104393	6558	13824	13823	4116	104394	13821	13819	13818
	0	0	0	0	0	0	0	0	0	0	0	0	0	0	12234	zyba_2_14_n01490	104590	12155	104589	104588	135751	104587	7184	13181	37146	134	14235	12155	55	7184	12155
	4014	14674	174	55	14675	3199	104137	7261	10054	15	12871	225	13884	3841	13883	zyba_2_2_b00600	10010	36605	12732	11260	12731	6584	2080	37045	7669	15	3184	12664	6614	348	15130
	0	0	0	0	0	0	0	0	22208	12155	735	12155	104642	104641	12155	zyba_2_33_ag00120	104640	1213	0	0	0	0	0	0	0	0	0	0	0	0	0
Z. bailii CLIB213	135863	20797	135862	635	13838	13837	13836	13835	13834	5643	13833	104391	15510	13829	20246	zyba_3_2_b02230	46332	13828	104392	1464	7843	13826	104393	6558	13824	13823	4116	104394	13821	13819	13818
	4014	14674	174	55	14675	3199	104137	7261	10054	15	12871	225	13884	3841	13883	zyba_3_3_c03460	10010	36605	12732	11260	12731	6584	2080	37045	7669	15	3184	12664	6614	348	15130
Z. rouxii CBS 732	12852	570	655	12851	12850	9574	12849	12848	104745	83568	12846	174	18251	949	6478	zyro_1_a04114g	10010	36605	12732	11260	12731	6584	2080	37045	7669	15	3184	12664	6614	348	15129
	14273	104796	4008	14271	18958	14270	12954	12953	135678	12950	12142	21974	8053	4430	12949	zyro_1_b14762g	12948	12947	3817	12946	1366	12606	12945	7457	97400	12942	12940	104256	2564	7459	15893
T. delbrueckii CBS 1146	4508	12854	12853	12852	570	655	12851	12850	9574	12849	12848	83568	12846	174	99300	tode_1_d04040	10010	12732	11260	12731	6584	2080	37045	7669	15	3184	12664	6614	348	15129	65805
L. kluyvery CBS 3082	70687	7100	9725	65466	55	55	2080	6584	12731	11260	12732	134	36605	10802	70686	lakl_1_c11616g	10010	6478	949	18251	174	12846	12847	12848	12849	9574	12850	12851	655	570	12852
	13371	1759	15	71172	13709	1072	71171	13373	13374	13375	13376	71170	13378	16550	54775	lakl_1_h21010g	71169	71168	13384	8144	13385	14827	13396	14190	67495	12357	12356	71167	71166	12355	12354
L. thermotolerans CBS 6340	12384	375	1208	2278	9414	12385	12386	12389	71216	10785	9209	9511	18251	949	6478	lath_1_a01914g	36605	12732	11260	12731	6584	2080	65466	9725	7100	71215	71214	12730	16803	1575	12729
L. waltii NCYC 2644	1208	2278	9414	12385	12385	12386	114064	12389	72647	10785	72648	9209	9511	72649	72650	lawa_1_23.5161	36605	12732	72651	114065	11260	114066	12731	72652	114067	114068	6584	72653	2080	72654	65466
K. aestuarii ATCC 18862	0	0	0	0	0	0	0	0	0	0	0	0	0	6478	67288	klae_1_14_n00120	10010	134	12732	11260	12731	2080	6584	225	12983	8661	67289	67290	12986	12987	12988
K. lactis CLIB210	12908	485	18191	265	15	13857	13858	13859	67444	13861	113096	9785	67341	13863	3986	klla_1_d03432g	10010	134	12732	11260	12731	2080	6584	225	12983	8661	67289	68137	67290	12986	12987
K. marxianus var. *marxianus* KCTC 17555	68416	12908	485	18191	265	15	13857	13858	13859	67444	13861	9785	67341	13863	3986	klma_1_1_a01880	10010	134	12732	11260	12731	2080	6584	225	12983	8661	67289	68415	67290	12986	12987
K. wickerhamii UCD 54-210	0	0	0	0	0	0	0	0	11521	3969	18251	949	6478	67288	68963	klwi_1_33_ag00170	68964	10010	134	12732	11260	12731	2080	6584	225	12983	8661	67289	68965	67290	12986
A. aceri	174	14793	7000	11212	14792	6284	14791	14790	14789	14788	14787	14786	14785	14784	134	asac_1_6_f03560	10010	14783	5571	14782	2819	1188	14781	14780	9670	14779	3093	12155	12155	12155	12155
E. cymbalariae CLIB210	4493	54920	7019	400	1786	12490	13396	13397	5174	140	13398	2080	6584	12732	134	ercy_1_3604	10010	13447	13448	13449	2681	13450	13451	6929	13452	54919	13454	13456	13457	15	13458
E. gossypii ATCC 10895	7332	174	14793	7000	11212	14792	6284	14791	14790	14789	14788	14787	14786	14784	134	ergo_1_abr125c	10010	14783	5571	14782	2819	1188	14781	14780	9670	14779	3093	12155	105	14778	9579

Supplementary figure S2: Phylogenetic tree of the Pdr18 and Snq2 orthologs from the left sub-lineage of figure 4, encoded in the genomes of the post-WGD species. (A) Radial phylogram showing the amino acid sequence similarity distances between the protein homologs of Pdr18 and Snq2 under study **(B)** Cladogram showing the tree topology.

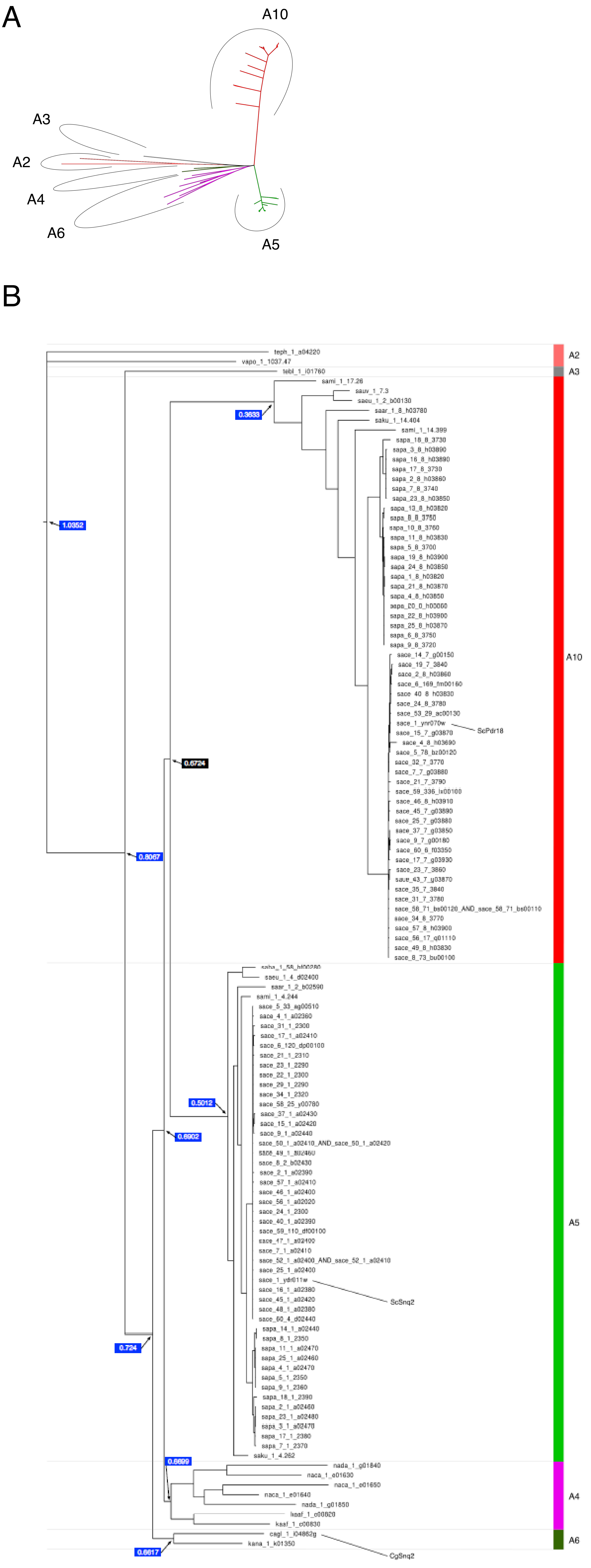

Supplement: Supplementary file 1 [file Data_Sheet_1.PDF]
