# Supplementary material for: The Paralogous Genes PDR18 and SNQ2, Encoding Multidrug Resistance ABC Transporters, Derive From a Recent Duplication Event, PDR18 Being Specific to the Saccharomyces Genus
Source: Front Genet. 2018 Oct 15;9:476. doi: 10.3389/fgene.2018.00476 (PMC6196229; doi:10.3389/fgene.2018.00476)
Supplement: Supplementary file 2 [file Data_Sheet_2.PDF]

Supplementary Table S1. Saccharomycetaceae yeast strains examined in this work, belonging to (A) *Saccharomyces* genus or (B) non-*Saccharomyces* genera. List of Saccharomycetaceae yeast strains genomes analysed in this study, corresponding source and annotation tool, and number of Snq2/Pdr18 homologs, Snq2 orthologs and Pdr18 orthologs identified. Pre-whole genome duplication (WGD) species are highlighted in grey. SGD Saccharomyces Genome Database (http://www.yeastgenome.org/), Genbank (http://www.ncbi.nlm.nih.gov/genome/browse/), Sanger Center (http://www.sanger.ac.uk/research/projects/genomeinformatics/sgrp.html), YGOB Yeast Gene Order Browser (http://ygob.ucd.ie/), YGAP Yeast Genome Annotation Pipeline (http://wolfe.ucd.ie/annotation/), DOE Joint Genome Institute (https://genome.jgi.doe.gov/saccharomycotina/saccharomycotina.info.html)

Supplementary Table S1A

Species	Strain	Strain acronym	Snq2 and Pdr18 homologues	Snq2 orthologues	Pdr18 orthologues	Annotation software/ site	Genome source
Saccharomyces cerevisiae	S288c	sace_1	2	1	1	SGD	SGD
	AWRI796	sace_2	2	1	1	YGAP	SGD
	cen.pk113-7d	sace_3	1	1	nd	YGAP	SGD
	FostersB	sace_4	2	1	1	YGAP	SGD
	FostersO	sace_5	2	1	1	YGAP	Genbank
	JAY291	sace_6	2	1	1	YGAP	Genbank
	BC187	sace_7	2	1	1	YGAP	Sanger centre
	Lalvin QA23	sace_8	2	1	1	YGAP	Genbank
	M3707	sace_9	2	1	1	YGAP	DOE JGI
	M3836	sace_10	2	1	1	YGAP	DOE JGI
	M3837	sace_11	2	1	1	YGAP	DOE JGI
	M3838	sace_12	2	1	1	YGAP	DOE JGI
	M3839	sace_13	2	1	1	YGAP	DOE JGI
	RM11-1a	sace_14	1	0	1	YGAP	Broad Institute
	273614N	sace_15	2	1	1	YGAP	Sanger centre
	322134S	sace_16	2	1	1	YGAP	Sanger centre
	378604X	sace_17	2	1	1	YGAP	Sanger centre
	DBVPG1106	sace_18	2	1	1	YGAP	Sanger centre
	DBVPG1373	sace_19	2	1	1	YGAP	Sanger centre
	DBVPG1788	sace_20	2	1	1	YGAP	Sanger centre
	DBVPG1853	sace_21	2	1	1	YGAP	Sanger centre
	DBVPG6040	sace_22	2	1	1	YGAP	Sanger centre
	DBVPG6044	sace_23	2	1	1	YGAP	Sanger centre
	DBVPG6765	sace_24	2	1	1	YGAP	Sanger centre
	K11	sace_25	2	1	1	YGAP	Sanger centre
	L 1374	sace_26	2	1	1	YGAP	Sanger centre
	L 1528	sace_27	2	1	1	YGAP	Sanger centre
	NCYC110	sace_28	2	1	1	YGAP	Sanger centre
	NCYC361	sace_29	2	1	1	YGAP	Sanger centre
	SK1	sace_30	2	1	1	YGAP	Sanger centre
	UWOPS03 461 4	sace_31	2	1	1	YGAP	Sanger centre
	UWOPS05 217 3	sace_32	2	1	1	YGAP	Sanger centre
	UWOPS05 227 2	sace_33	2	1	1	YGAP	Sanger centre
	UWOPS83 787 3	sace_34	2	1	1	YGAP	Sanger centre
	UWOPS87 2421	sace_35	2	1	1	YGAP	Sanger centre
	Y9	sace_36	2	1	1	YGAP	Sanger centre
	Y12	sace_37	2	1	1	YGAP	Sanger centre
	Y55	sace_38	2	1	1	YGAP	Sanger centre
	YIIc17 E5	sace_39	2	1	1	YGAP	Sanger centre
	YJM975	sace_40	2	1	1	YGAP	Sanger centre

Supplementary Table 1A (continued)

Species	Strain	Strain acronym	Snq2 and Pdr18 homologues	Snq2 orthologues	Pdr18 orthologues	Annotation software/ site	Genome source
Saccharomyces cerevisiae	YJM978	sace_41	2	1	1	YGAP	Sanger centre
	YJM981	sace_42	2	1	1	YGAP	Sanger centre
	YPS128	sace_43	2	1	1	YGAP	Sanger centre
	YPS606	sace_44	2	1	1	YGAP	Sanger centre
	YS2	sace_45	2	1	1	YGAP	Sanger centre
	YS4	sace_46	2	1	1	YGAP	Sanger centre
	YS9	sace_47	2	1	1	YGAP	Sanger centre
	Sigma1278b	sace_48	1	1	0	YGAP	SGD
	vin13	sace_49	2	1	1	YGAP	SGD
	VL3	sace_50	1	1	0	YGAP	SGD
	w303	sace_51	1	1	0	YGAP	SGD
	YJM789	sace_52	2	1	1	YGAP	Genbank
	EDRL	sace_53	2	1	1	YGAP	DOE JGI
	BY4741	sace_54	2	1	1	YGAP	SGD
	BY4742	sace_55	2	1	1	YGAP	SGD
	EC1118	sace_56	2	1	1	YGAP	Genbank
	Kyokai7	sace_57	2	1	1	YGAP	SGD
	T7	sace_58	2	1	1	YGAP	SGD
	YB210	sace_59	2	1	1	YGAP	DOE JGI
	ZTW1	sace_60	2	1	1	YGAP	Genbank
Saccharomyces paradoxus	reference consensus	sapa_1	2	1	1	YGAP	Sanger centre
	A4	sapa_2	2	1	1	YGAP	Sanger centre
	A12	sapa_3	2	1	1	YGAP	Sanger centre
	CBS432	sapa_4	2	1	1	YGAP	Sanger centre
	CBS5829	sapa_5	2	1	1	YGAP	Sanger centre
	DBVPG4650	sapa_6	2	1	1	YGAP	Sanger centre
	DBVPG6304	sapa_7	2	1	1	YGAP	Sanger centre
	IFO1804	sapa_8	2	1	1	YGAP	Sanger centre
	KPN3828	sapa_9	2	1	1	YGAP	Sanger centre
	KPN3829	sapa_10	2	1	1	YGAP	Sanger centre
	N 17	sapa_11	2	1	1	YGAP	Sanger centre
	N 43	sapa_12	2	1	1	YGAP	Sanger centre
	N 44	sapa_13	2	1	1	YGAP	Sanger centre
	N 45	sapa_14	2	1	1	YGAP	Sanger centre
	Q31 4	sapa_15	2	1	1	YGAP	Sanger centre
	UFRJ50791	sapa_16	2	1	1	YGAP	Sanger centre
	UFRJ50816	sapa_17	2	1	1	YGAP	Sanger centre
	UWOPS91 917 1	sapa_18	2	1	1	YGAP	Sanger centre
	W7	sapa_19	2	1	1	YGAP	Sanger centre
	Y6 5	sapa_20	2	1	1	YGAP	Sanger centre
	Y7	sapa_21	2	1	1	YGAP	Sanger centre
	Y8 5	sapa_22	2	1	1	YGAP	Sanger centre
	YPS138	sapa_23	2	1	1	YGAP	Sanger centre
	Z1	sapa_24	2	1	1	YGAP	Sanger centre
	Z1 1	sapa_25	2	1	1	YGAP	Sanger centre

Supplementary Table 1A (continued)

Species	Strain	Strain acronym	Snq2 and Pdr18 homologues	Snq2 orthologues	Pdr18 orthologues	Annotation software/ site	Genome source
Saccharomyces mikatae	IFO 1815	sami_1	3	1	1	YGOB	YGOB
Saccharomyces kudriavzevii	IFO 1802	saku_1	2	1	1	YGOB	YGOB
Saccharomyces arboricola	H-6	saar_1	2	1	1	YGAP	Genbank
Saccharomyces eubayanus	FM1318	saeu_1	2	1	1	YGAP	Genbank
	CBS12357	saeu_2	2	1	1	YGAP	Genbank
Saccharomyces bayanus (HYBRID)	623-6C	saba_1	1	1	0	YGAP	SGD
	MCYC 623	saba_2	0	0	0	YGAP	SGD
Saccharomyces uvarum	CBS 7001	sauv_1	1	0	1	YGOB	YGOB

Supplementary Table 1B

Species	Strain	Strain acronym	Snq2 orthologues	Annotation software/site	Genome source
Kazachstania africana	CBS 2517	kaaf_1	2	YGOB	YGOB
Kazachstania naganishii	CBS 8797	kana_1	1	YGOB	YGOB
Naumovozyma castellii	CBS 4309	naca_1	3	YGOB	YGOB
	NRRL Y-12630	naca_2	1	YGAP	SGD
Naumovozyma dairenensis	CBS 421	nada_1	2	YGOB	YGOB
Candida glabrata	CBS138	cagl_1	1	YGOB	YGOB
	CCTCC M202019	cagl_2	1	YGAP	Genbank
Tetrapisispora blattae	CBS 6284	tebl_1	2	YGOB	YGOB
Tetrapisispora phaffii	CBS 4417	teph_1	2	YGOB	YGOB
Vanderwaltozyma polyspora	DSM 70294	vapo_1	2	YGOB	YGOB
Zygosaccharomyces bailii	IST302	zyba_2	4	MIPS	EBI
	CLIB 213	zyba_3	2	YGAP	Genbank
Zygosaccharomyces rouxii	CBS 732	zyro_1	2	YGOB	YGOB
Torulaspora delbrueckii	CBS 1146	tode_1	1	YGOB	YGOB
Lachancea kluyvery	CBS 3082	lakl_1	2	YGOB	YGOB
Lachancea thermotolerans	CBS 6340	lath_1	1	YGOB	YGOB
Lachancea waltii	NCYC 2644	lawa_1	1	YGOB	YGOB
Kluyveromyces aestuarii	ATCC 18862	klae_1	1	YGAP	Genbank
Kluyveromyces lactis	CLIB210	klla_1	1	YGOB	YGOB
Kluyveromyces marxianus var. marxianus	KCTC 17555	klma_1	1	YGAP	Genbank
Kluyveromyces wickerhamii	UCD 54-210	klwi_1	1	YGAP	Genbank
Ashbya aceri	-	asac_1	1	YGAP	Genbank
Eremothecium cymbalariae	DBVPG 7215	ercy_1	1	YGOB	YGOB
Eremothecium gossypii	ATCC 10895	ergo_1	1	YGOB	YGOB
